# Individual wearable air purifier protects against pollen, house dust mite, and cat allergens: Report from an allergen exposure chamber 

**DOI:** 10.5414/ALX02473E

**Published:** 2024-03-21

**Authors:** Karl-Christian Bergmann, Teresa Hartung, Torsten Zuberbier

**Affiliations:** 1 Institute of Allergology, Charité-Universitätsmedizin Berlin, and; 2 European Center Allergy Research Foundation, Berlin, Germany

**Keywords:** allergen avoidance, air purifier, wearable device, cat allergens, house dust mite allergens, birch pollen

## Abstract

Purpose: Evaluation of a new individual wearable air purifier (Respiray Wear A+) for birch pollen, house dust mite (HDM), and cat-allergic rhinoconjunctivitis (ARC) patients in a standardized allergen exposure chamber (AEC). Materials and methods: Eligible allergic patients were exposed to birch pollen, HDM raw material, and cat allergen in an AEC for 60 minutes without (V1) and with (V3) the use of the Respiray device. Nasal, ocular, bronchial, and other symptoms were rated by the patients every 10 minutes, and their wellbeing, peak nasal inspiratory flow (PNIF), and lung function parameters were assessed every 30 minutes. The primary endpoint was the change in the median of the total symptom score (TSS) at V3 compared to V1 at 60 minutes of exposure. The secondary endpoints consisted of the total nasal symptom score (TNSS) and total eye symptom score (TESS). Results: 23 patients with birch pollen allergy, 37 patients with HDM allergy, and 41 patients with cat allergy were included in the analysis. Significant reduced symptom scores of ~ 49% were observed when using Respiray Wea A+ under birch pollen exposure (p < 0.05) in the primary endpoint TSS (V3 2.43 compared to V1 4.78). An 48% reduction of symptoms was seen in TSS in case of HDM exposure (V3 3.59; V1 6.92, (t-test: p < 0.01)) and the highest reduction of TSS (60%) under Respiray A+ using cat allergens (V3 2.95, V1 7.44, (t-test p < 0.01) after 60 minutes of exposure. The personal wellbeing revealed clinically meaningful improvements over time in all three studies which manifested in a lower symptom increase during the final allergen exposures. Conclusion: The individual wearable air purifier Respiray Wear A+ protects significantly against airborne pollen, HDM, and cat allergens and may be a very useful device for avoiding indoor allergens in a new way.

## Introduction 

Depending on the geographic location and age of the patients, allergic rhinitis (AR) and allergic rhinoconjunctivitis (ARC) affect up to 10 – 40% of the world’s population [1]. The prevalence of sensitization to airborne outside or indoor allergens reaches even higher values. 

AR presents especially with nasal congestion, sneezing, nasal pruritus, and nasal discharge [2]. In Europe, the major cause for seasonal allergic rhinitis (SAR) is outdoor pollen. Typical indoor allergens such as house dust mites (HDM) and animal dander, e.g., cat allergens, are the most common triggers for perennial allergic rhinitis (PAR). 

SAR and PAR are known to result in a decreased quality of life, sleep disorders, missing days at work or school, decreased productivity, and eventually cause a direct and indirect economic burden of billions of Euro for the society [3]. 

Hence, there is a great need for developing new options for sensitized individuals to avoid the respective allergens outdoors and indoors. Allergen avoidance is recognized as the first-line method to prevent symptoms with significant coverage in the treatment guidelines [4]. Many allergy sufferers rely on medications and look for non-medical options to avoid symptoms [5, 6]. 

Allergen exposure chambers (AECs) can be used as a tool for controlled exposure to allergenic and non-allergenic airborne particles in an enclosed environment, in order to (i) characterize the pathological features of respiratory diseases and (ii) to contribute to and accelerate the clinical development of pharmacological treatments including approved allergen immunotherapy [7]. AECs can also be used to prove efficacy of avoidance methods for allergic diseases of the respiratory tract. 

This study aims to evaluate a newly developed, wearable, air-purifying device for individuals, called Respiray Wear A+ (Respiray OÜ, Tallin, Estonia), for protection against AR and ARC symptoms due to birch pollen, HDM, and cat allergens. 

## Materials and methods 

### Subjects 

The eligibility criteria for study participation were: birch pollen, HDM, or cat allergy, with ARC symptoms for ≥ 2 years according to the ARIA guidelines [1], ages 18 – 65 years, skin prick test (SPT) response (wheal diameter ≥ 3 mm) to birch pollen, HDM (*Dermatophagoides pteronyssinus* (*D. pt.*) and/or *Dermatophagoides farinae* (*D. f.*)), and cat allergen, and a minimum total nasal symptom score (TNSS) ≥ 3 during the first provocation in the AEC. 

The main exclusion criteria were: sublingual or subcutaneous allergen immunotherapy (SLIT/SCIT) during the last 2 years, severe or uncontrolled asthma during 3 months before screening, FEV_1_ < 80% predicted before allergen exposure, relevant infectious or severe chronic diseases or contraindication to adrenaline and/or other rescue medication, simultaneous intake of anti-allergic medication prior to screening process and exposure in the AEC. Table 1 summarizes the characteristics of participants. 

### AEC 

The GA^2^LEN AEC (ECARF) is a mobile flexible chamber made of two connected standard 24 feet (7.32 m) high cube containers [8, 9]. In the standardized and validated chamber, the exposure in V1 and V3 is performed with 8,000 birch pollen/m^3^ (Stallergenes Greer, Lenoir, NC, USA), 300 μg/m^3^ HDM raw material (whole culture mite: D. pt. and D. f. body and excrement allergen 50% : 50%) (Allergon AB, Ängelholm, Sweden), or 400 µg/m^3^ cat allergen (particles, Stallergenes Greer) for 60 minutes at 20 °C and 55% relative humidity. The degree of exposure is calculated by the number of birch pollen, HDM particles, and cat allergen particles and the amount of allergen in the allergen materials. 

Before the start of the test, the subjects underwent an acclimatization phase of 20 minutes without exposure in the chamber. 

### Outcome parameters 

Each symptom was evaluated by the subjects on a scale from 0 to 3 (no, mild, moderate, or severe symptoms) and summed up to give a TNSS (runny, sneezing, itchy, and blocked nose), total eye symptom score (TESS) (itchy, watery eyes, and gritty feeling), and total symptom score (TSS), which is the sum of TNSS and TESS with a maximum score of 21. The primary endpoint was the change in TSS at 60 minutes of exposure to birch pollen, HDM raw material, and cat allergen in the AEC at visit V3 compared to visit V1. 

Changes in personal well-being (visual analogue scale (VAS): 0 = very good to 10 = very bad) were recorded using a VAS before and every 30 minutes during the exposure. Forced expiratory volume in 1 second (FEV_1_;) (EasyOne Spirometer, ndd Medizintechnik AG, Zürich, Switzerland) was performed before and after exposure. To record late phase reactions (LPRs) or adverse events (AEs) related to the exposure, participants received a safety call 24 hours after each exposure. 

### Statistical analysis 

Percent changes between AEC visits were calculated by first calculating the mean of values measured during V1 and V3 separately over all patients at 60 minutes. The following equation was employed to retrieve percent changes: ((mean V3 – mean V1)/mean V1) × 100. Mean values and percent changes will be presented. 

At first, the data were tested for their distribution. In case of a normal distribution a paired t-test was performed. A t-test compares the mean/average of two groups (in our case baseline and V3) and discovers whether the difference is by random chance. A t-test can be either calculated with independent samples or with paired samples. Paired samples consist of matched pairs of for example one group of units that has been tested twice, which is the case in our study. 

All analyses were performed with R version 4.3.1 (2023-06-16) [10] using the packages “graphics” (v4.3.1) for mixed effects modeling and “stats” (v4.3.1) for estimating p-values of fixed effects [11]. The data are depicted in linear mixed effects models. 

### Course of study 

Each study (Table 2) was divided into a screening phase (V0) for the selection of suitable subjects, a baseline exposure (V1) including follow-up phone call (V2), and a further final exposure (V3) with the associated follow-up phone call (safety phone call, V4). 

### Air purifier Respiray Wear A+ 

Respiray Wear A+ is personal air purifier that is worn around the neck. The device works by taking in air from below, purifying it with a HEPA filter, and then blowing clean air out from the top. This creates a clean air buffer zone around the user’s head, providing them with clean air. 

**Figure 1.**
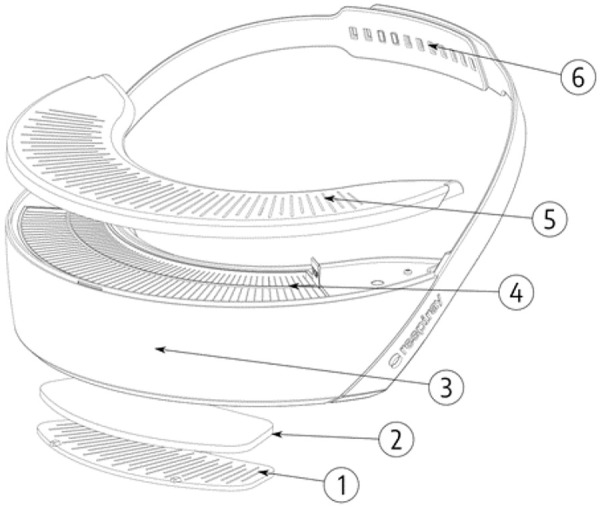
Components of Respiray Wear A+. 1 = Air is drawn into the device through the intake grille. 2 = Large particles are filtered using the pre-filter. 3 = The body of the device houses fans, battery, and electronics. 4 = The HEPA filter blocks allergens and allows only clean air to pass through. 5 = Clean air is blown out from the output grilles. 6 = The device is worn on the user’s chest using straps.

The main components of the Respiray Wear A+ are depicted in Figure 1. 

The device has been optimized to minimize the mixing of ambient air with clean air from the device within the buffer zone. Multiple methods were employed to attain this objective, such as laser sheet visualization of airflow patterns surrounding the device and the user, aerosol chamber testing with dolls, and computational fluid dynamics (CFD) simulations. The reduced severity of symptoms when using the Respiray Wear A+ suggests that the concentration of allergens in the air has been reduced as a result of the filter in the device. 

**Figure 2.**
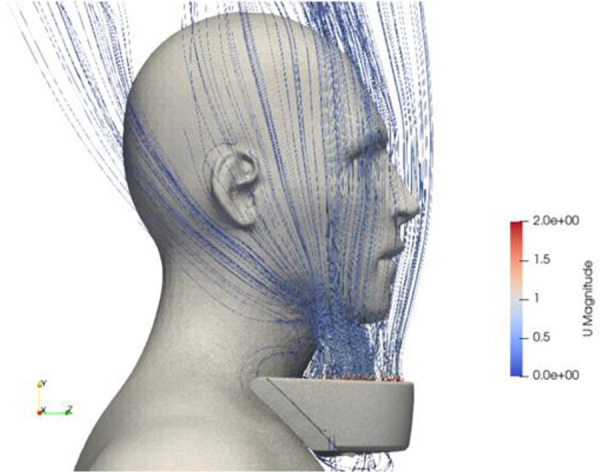
Capture from a CFD simulation depicting the streamlines exiting the device.

Figure 2 demonstrates the clean air delivery from the device to the user. 

The final design of the device has two operational modes: a “normal mode” which provides 70 L/min of clean air, and a “high-speed mode” which provides 130 L/min of clean air. The high-speed mode offers increased protection in situations where the clean air buffer zone may be disrupted due to fast user movement or gusts of wind. In the study, the normal mode was used. 

## Results 

### Efficacy 

At V3, after 60 minutes of birch pollen, HDM, or cat allergen exposure, the median TSS described a significant difference of 49%, 48%, and even 60% for cat allergen due to the use of Respiray Wear A+ in comparison to V1 (exposure without the device), respectively. Table 3 summarizes the results of the TSS for all three allergens. 

**Figure 3.**
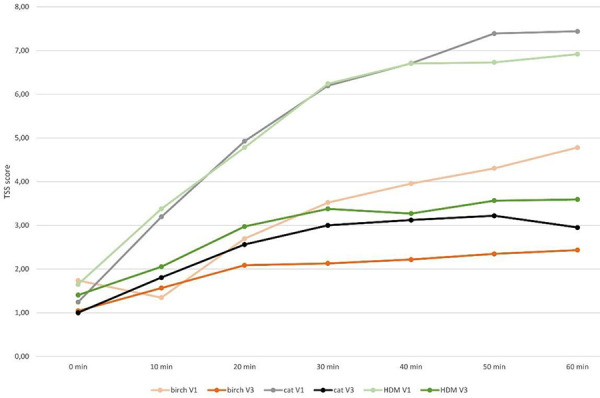
Mean values of the sums of total symptom score (TSS) for V1 (without Respiray) and V3 (with Respiray) during exposure to birch, house dust mite, and cat allergens.

**Figure 4.**
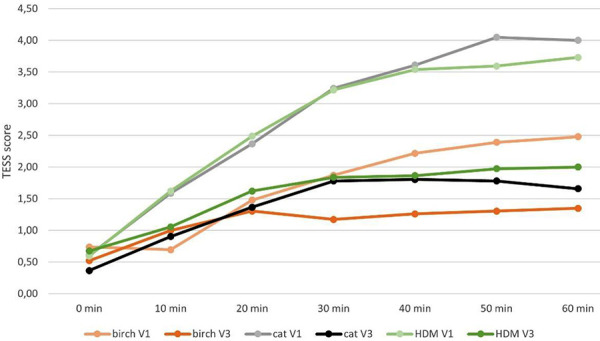
Mean values of the sums of total eye symptom score (TESS) for V1 (without Respiray) and V3 (with Respiray) during exposure to birch, house dust mite, and cat allergens.

**Figure 5.**
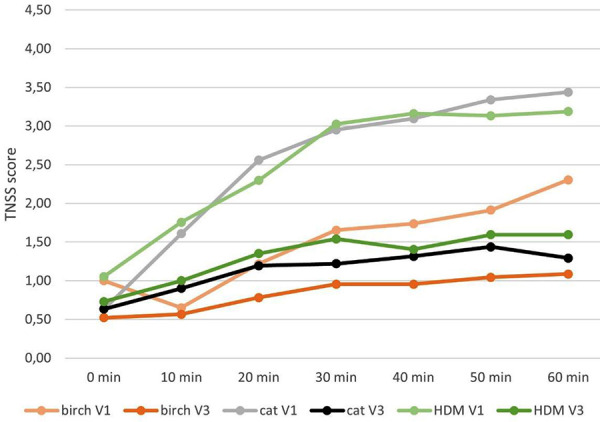
Mean values of the sums of total nose symptom score (TNSS) for V1 (without Respiray) and V3 (with Respiray) during exposure to birch, house dust mite, and cat allergens.

Further exploratory endpoints included analysis of the temporal development of the TSS during 60 minutes of exposure. We detected a relevant decreased symptom severity when using the Respiray Wear A+ which is reflected in a lower rate of increase in symptoms over time (Figures 3, 4, 5). 

For all tested allergens, the improvement rates for the TESS were over 45% (Table 4); however, even higher improvement rates of over 50% were observed for the TNSS values (Table 5). 

No relevant differences between V1 and V3 were observed for peak nasal inspiratory flow (PNIF), peak expiratory flow (PEF), and spirometry parameters. 

**Figure 6.**
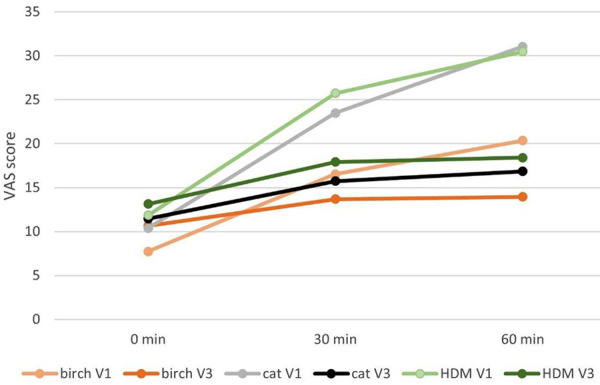
Mean values of the visual analog scale scores at minutes 0, 30, and 60 at baseline and final exposure to all different allergens without Respiray (V1) and with Respiray (V3).

The rate of improvement in well-being, measured using the visual analogue scale (VAS), increased clearly when using Respiray Wear A+, as summarized in Table 6. However, statistical significance could not be detected (Figure 6).[Table Table7]

During the safety call that took place 24 hours after birch pollen exposure without Respiray, 12/23 subjects (52%) described late reactions. When using Respiray it was only 7/23 subjects (30%). Following HDM exposure without Respiray, 20 of the 37 subjects (54%) described late reactions, while when using Respiray late reactions occurred in 13/37 (35%) subjects. After cat allergen exposure 18/41 subjects (44%) reported late reactions without Respiray, and only 5/41 (12%) with Respiray. Obstructed nose, mild cough or mild dyspnea, irritated or sore throat have been described as symptoms in a late reaction. This indicates an increase in tolerability to birch pollen, HDM, and cat allergens during exposure in the AEC when using the device. 

### Safety 

No AEs were reported following the allergen exposures without and with the Respiray Wear A+ device at V4. 

## Discussion 

The continued high prevalence of allergic diseases in Western industrialized nations combined with the limited options for causal therapy emphasize the necessity for evidence-based primary prevention, including effective avoidance methods. 

The goal of avoidance options is to prevent allergens from coming into contact with the respiratory tract mucosa and therefore inhibiting the occurrence of allergic symptoms. At the same time, avoidance methods have a medication-sparing effect and many patients prefer non-pharmacological measures to prevent allergic symptoms [6]. As an example, the use of air purifiers with HEPA filters significantly reduces medication requirements for patients with HDM-induced allergies including asthma [12], and the quality of life improved in studies of air purifiers in asthmatics [13]. 

However, allergen avoidance is a controversial issue because it is difficult for the patients to fully implement these methods, and the level of evidence is not very high [4]. 

Controlling exposure to outdoor allergens, e.g., pollen, is much more difficult than controlling exposure to indoor allergens. 

Various methods have been described for pollen avoidance in AR patients with pollen sensitivity, and face mask usage during pollen seasons is one of the recommended avoidance methods [14]. 

In a randomized, double‐blind, controlled clinical trial with active and inactive versions of an air purifier, patients with AR who were sensitive to *Artemisia* pollen had reduced symptoms when using air filtration at night [15]. 

Evidence supporting the effect of individual protective measures (IPMs) on air pollution is also relatively scarce. One systematic literature review was carried out to examine the impact of portable air purifiers (PAPs) on indoor air quality (PM_2.5_) and health, focusing on adults and children in indoor environments (homes, schools, and offices). All studies showed reductions in PM_2.5_ of between 22.6 and 92.0% with the use of PAPs when compared to the control [16]. 

Meta-analysis results could demonstrate a reduction in cardiopulmonary inflammation when using air purifiers versus the control groups (with sham/no filter), with a decrease in interleukin 6. A sub-group analysis for air purifiers as an IPM in developing countries reduced fractional exhaled nitric oxide. Therefore, air purifiers can serve as efficient IPMs not only against airborne allergens but also against air pollution [17]. 

To evaluate an “anti-airborne-allergen effect”, 23 adults with confirmed allergic rhinoconjunctivitis were exposed to birch pollen outside the birch pollen season, to HDM (n = 37), or to cat allergens (n = 41) in an exposure chamber for 60 minutes in a standardized manner without and with the individual wearable air purifier Respiray Wear A+. 

The device cleans the air from large particles using the pre-filter, and the HEPA filter blocks airborne allergens and allows only clean air to pass through. Clean air is blown out from the output grilles that surround the face and protect nose and eyes; only clean air can be inhaled leading to a clear reduction of nasal and conjunctival symptoms to all three tested allergens. 

The device reduced the TSS by ~ 50% in birch pollen-allergic subjects, by 48% in patients with HDM allergy, and by 60% in cat-allergic subjects. Most anti-allergy medications do not relieve the symptoms to this extent.. Allergen-specific immunotherapy – although the goal is not comparable to the use of filtered air – hardly achieves such an effect. 

We may assume that wearing the Respiray Wear A+ during the pollen season, especially during the peak pollen days with > 100 birch pollen/m^3^, will avoid a great part of the occurrence of nasal and ocular symptoms. The use of anti-allergic medications may be unnecessary. 

It can also be assumed that not only birch pollen but also grass pollen and herb pollen are effectively filtered through the Respiray device and can therefore lead to no or significantly fewer symptoms. 

Symptoms can also be avoided by using the device for certain types of outdoor work during the pollen season, e.g., agricultural work (harvesting, stable work, use of combine harvesters, etc.). Using the device outdoors will depend on wind conditions. In calm air, the filter effect will be good, stronger crosswinds will reduce the effectiveness. We have not investigated this. 

The protective effects during exposure to indoor allergens (mites and cat allergens) in relatively high concentrations for persons with AR or allergic rhinoconjunctivitis demonstrates the wide range of possible applications in the daily life. It has been shown that stationary air purifiers significantly prevent early and late asthmatic responses among cat-allergic asthmatics during cat allergen exposure [18], but individual highly mobile air purifiers such as the Respiray Wear A+ provide more freedom from allergens. 

Cat allergy sufferers who do not have an animal at home often cannot visit family members or friends because they have a cat or dog. Again, a good way to avoid symptoms for a few hours would be possible with the help of the Respiray device. 

In addition, during home cleaning procedures, the Respiray Wear A+ can be recommended as an effective non-drug option for those allergic to mites or animal allergens. 

## Conclusion 

The investigation of the Respiray Wear A+ demonstrated clinically relevant effectiveness in protecting individuals with birch pollen, HDM, or cat allergies in the robust indoor setting of an AEC. The air-filtration effect of the Respiray device results in the protection of eyes, nose, and bronchi against airborne pollen, HDM, or cat allergens. 

This effect might protect allergic subjects while spending time in the nature, in indoor environments (homes, offices, schools, nursing homes), or during cleaning of rooms. The Respiray device would also allow cat-allergic subjects to visit their families who own a cat at home and still avoid allergy symptoms. 

This novel device might be able to avoid allergy symptoms without the need for medication. 

## Ethical approval and informed consent 

The study protocol was approved by the Ethics Committee of the Charité Berlin (EA1/221/22; EA1/265/22; EA1/266/22). All participants received detailed information from the supervising physician and provided their written informed consent to participate. They also agreed to the processing and storage of their data in accordance with the General Data Protection Regulation. 

The study was conducted in accordance with the Declaration of Helsinki and in compliance with all federal, regional, and local requirements. Informed consent was obtained from all participants. All data provided were pseudonymized to protect the privacy of the patients who participated in the study as mandated by the applicable laws and regulations. 

## Authors’ contributions 

KCB and TZ designed the study. Data acquisition was done by KCB and TZ. TH performed the statistical analysis. KCB and TZ evaluated and interpreted the data and drafted the manuscript. All authors reviewed the manuscript critically and approved the final version and its submission. 

## Funding 

This study was funded by Respiray OÜ, Moisa 4, 13522 Tallin, Estonia. 

## Conflict of interest 

The authors declare no conflict of interest. 

**Table 1.** Characteristics of probands at the studies with birch pollen, house dust mite, and cat allergens.

**Subject data**	**Birch pollen ** **(n = 23)**	**Dust mites ** **(n = 37)**	**Cat allergens ** **(n = 41)**
Age (years), mean (SD)	39.4 (12.5)	40.4 (13.6)	35.4 (11.8)
Female, n (%)	17 (73.9)	31 (83.8)	32 (78)
Male, n (%)	6 (26.1)	6 (16.2)	9 (22)
Weight (kg), mean (SD)	74.7 (27.8)	76.4 (20.4)	71,7 (13.6)
Prick test (mm), mean (SD)	9.3 (3.5)	7.5 (2.7)	6.4 (1.8)

**Table 2.** Flow chart of the entire study.

**At least 7 days prior to V1**	**Day 1**	**Day 2**	**Day 7**	**Day 8**
Screening V0	Baseline exposure without Respiray V1	Safety call V2	Exposure with Respiray V3	Safety call V4

**Table 3.** Total symptom score values V1 and V3 at the 60-minute time point are used as base of operation to determine the improvement rate in percentage. The p-value was calculated applying a t-test. „Improvement“ in the table means fewer symptoms using Respiray Wear A+ in comparison to symptom severity without a filter.

**Allergen**	**Mean value baseline (V1)**	**Mean value – with filter (V3)**	**Improvement rate (%)**	**p-value**
Birch	4.78	2.43	49	0.01149
House dust mite	6.92	3.59	48	0.00236
Cat	7.44	2.95	60	0.00308

**Table 4.** Total eye symptom scores for V1 and V3 at the 60-minute time point are used as base of operation to determine the improvement rate in percent. The p-value was calculated applying a t-test. „Improvement“ in the table means fewer symptoms using Respiray Wear A+ in comparison to symptom severity without a filter.

**Allergen**	**Mean value – baseline (V1)**	**Mean value – with filter (V3)**	**Improvement rate (%)**	**p-value**
Birch	2.48	1.35	46	0.03390
House dust mite	3.73	2.00	46	0.00519
Cat	4.00	1.66	59	0.00308

**Table 5.** Total nasal symptom scores for V1 and V3 at the 60-minute time point are used as base of operation to determine the improvement rate in percent. The p-value was calculated applying a t-test. „Improvement“ in the table means fewer symptoms using Respiray in comparison to symptom severity without a filter.

**Allergen**	**Mean value – baseline (V1)**	**Mean value – with filter (V3)**	**Improvement rate (%)**	**p-value**
Birch	2.30	1.09	53	0.00307
HDM	3.19	1.59	50	0.00098
Cat	3.44	1.29	62	0.00311

**Table 6.** Visual analog scale well-being values for V1 and V3 at the 60-minute time point are used as base of operation to determine the improvement rate in percent. The p-value was calculated applying a t-test. „Improvement“ in the table means fewer symptoms using Respiray Wear A+ in comparison to symptom severity without a filter.

**Allergen**	**Mean value – baseline (V1)**	**Mean value – with filter (V3)**	**Improvement rate (%)**	**p-value**
Birch	21	14	31	0.5192
House dust mite	31	18	40	0.2556
Cat	31	16	46	0.2565

**Table 7. Table7:** Total symptom score values for V1 and V3 over the measured time frame. The mean value of all investigated subjects as well as the standard deviation are depicted.

**Allergen**	**Moment**	**0 minutes**		**10 minutes**		**20 minutes**		**30 minutes**		**40 minutes**		**50 minutes**		**60 minutes**	
		**Mean**	**SD**	**Mean**	**SD**	**Mean**	**SD**	**Mean**	**SD**	**Mean**	**SD**	**Mean**	**SD**	**Mean**	**SD**
Birch	Baseline (V1)	1.74	2.80	1.35	1.07	2.70	1.49	3.52	2.00	3.96	2.31	4.30	2.84	4.78	3.22
Birch	With filter (V3)	1.04	1.02	1.57	1.24	2.09	1.47	2.13	1.22	2.22	1.38	2.35	1.61	2.43	1.56
House dust mite	Baseline (V1)	1.65	1.77	3.38	2.30	4.78	2.65	6.24	3.15	6.70	3.13	6.73	3.17	6.92	3.23
House dust mite	With filter (V3)	1.41	1.38	2.05	1.84	2.97	1.64	3.38	2.14	3.27	2.29	3.57	2.57	3.59	2.67
Cat	Baseline (V1)	1.24	1.48	3.20	1.85	4.93	2.60	6.20	2.85	6.71	2.87	7.39	3.53	7.44	3.65
Cat	With filter (V3)	1.00	1.18	1.80	1.47	2.56	1.66	3.00	1.97	3.12	2.14	3.22	2.66	2.95	2.48
